# Supplementary figures and images for: A Cell-Based Screen Identifies HDAC Inhibitors as Activators of RIG-I Signaling
Source: Front Mol Biosci. 2022 Feb 14;9:837610. doi: 10.3389/fmolb.2022.837610 (PMC8882870; doi:10.3389/fmolb.2022.837610)

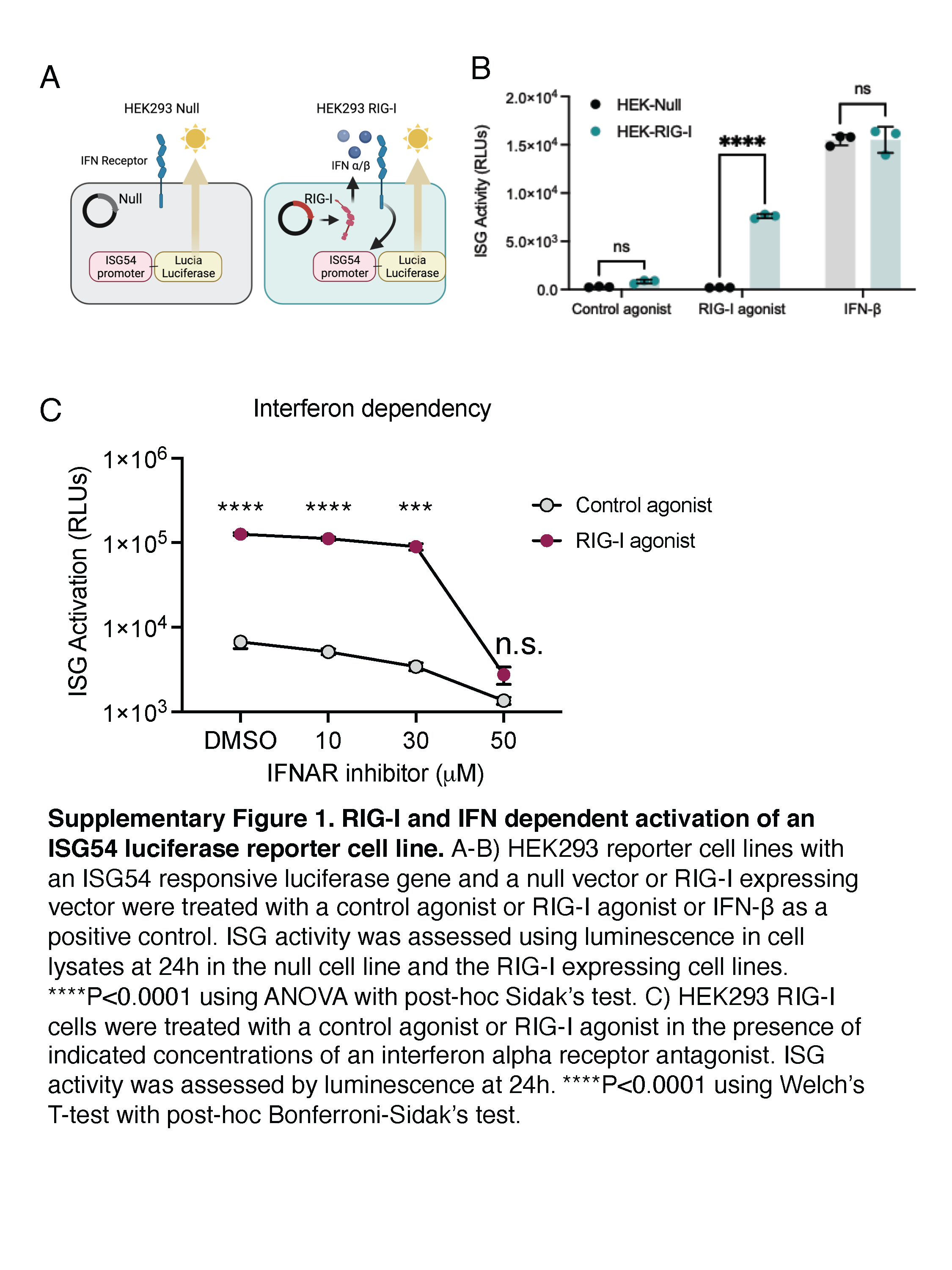

Supplement: Supplementary file 1 [file Image1.TIFF]

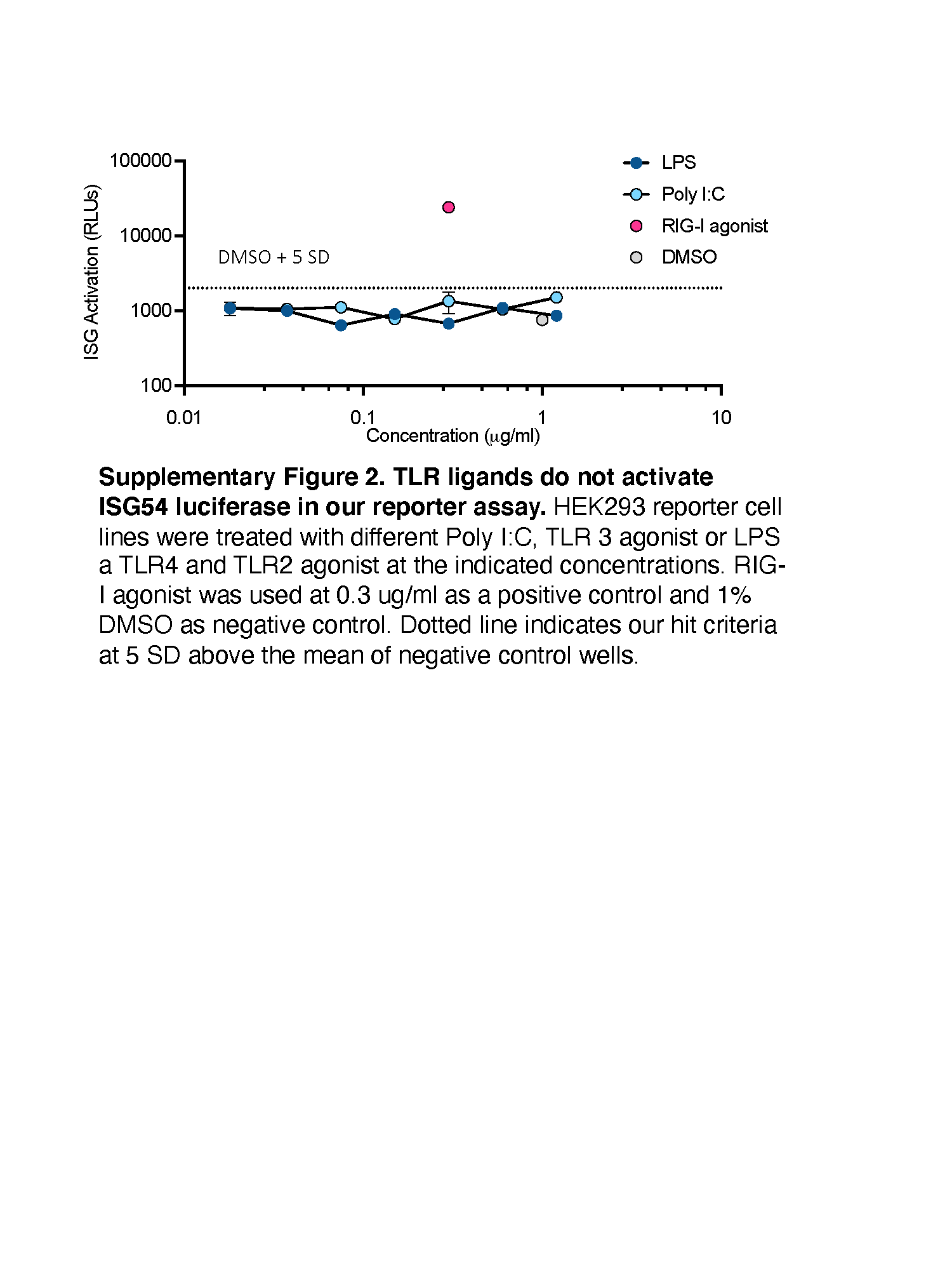

Supplement: Supplementary file 2 [file Image2.TIFF]
